# Supplementary figures and images for: Sequencing DNA with nanopores: Troubles and biases
Source: PLoS One. 2021 Oct 1;16(10):e0257521. doi: 10.1371/journal.pone.0257521 (PMC8486125; doi:10.1371/journal.pone.0257521)

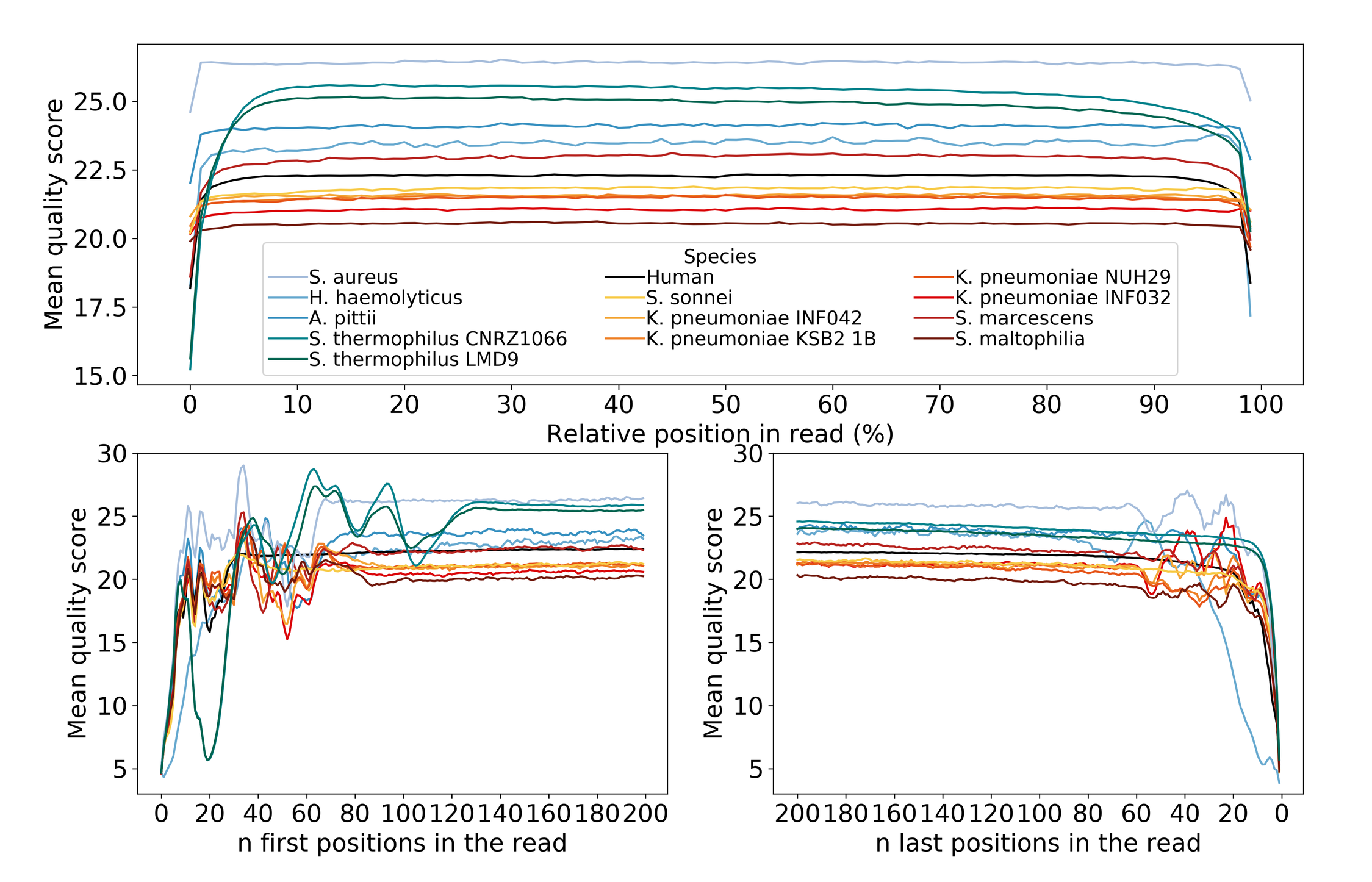

Supplement: S1 Fig — Top panel shows mean quality scores for relative position in read (gathered by %). Bottom panels zoom on both ends of reads, for the n first and last bases. (TIF) [file pone.0257521.s001.tif]

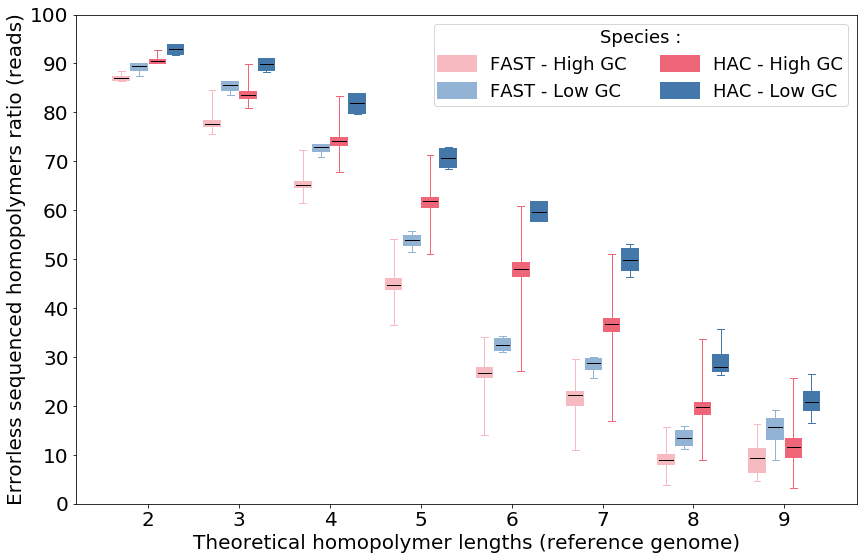

Supplement: S2 Fig — Results are split according to basecalling mode (HAC or FAST) and bacterial GC content (low or high). (TIF) [file pone.0257521.s002.tif]

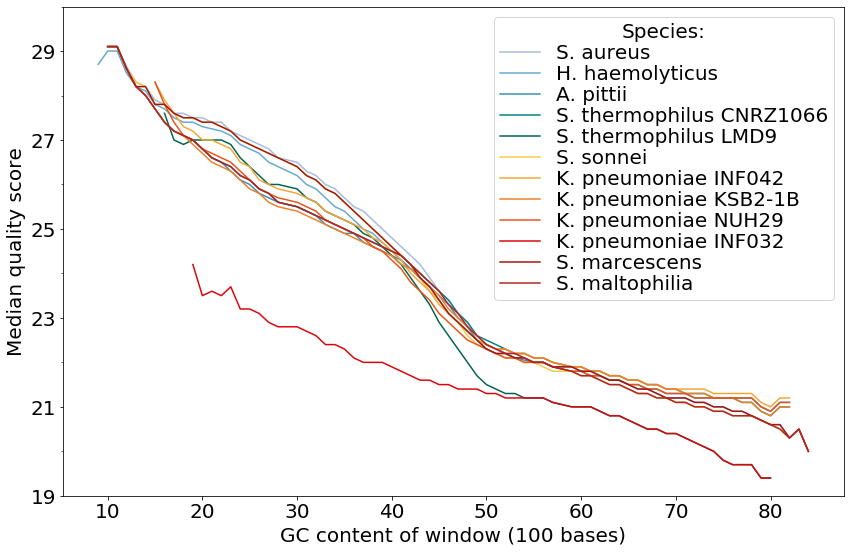

Supplement: S3 Fig — Note the drop of about 1.5 in quality around the central GC value. The outlier species is K. pneumoniae INF032. (TIF) [file pone.0257521.s003.tif]

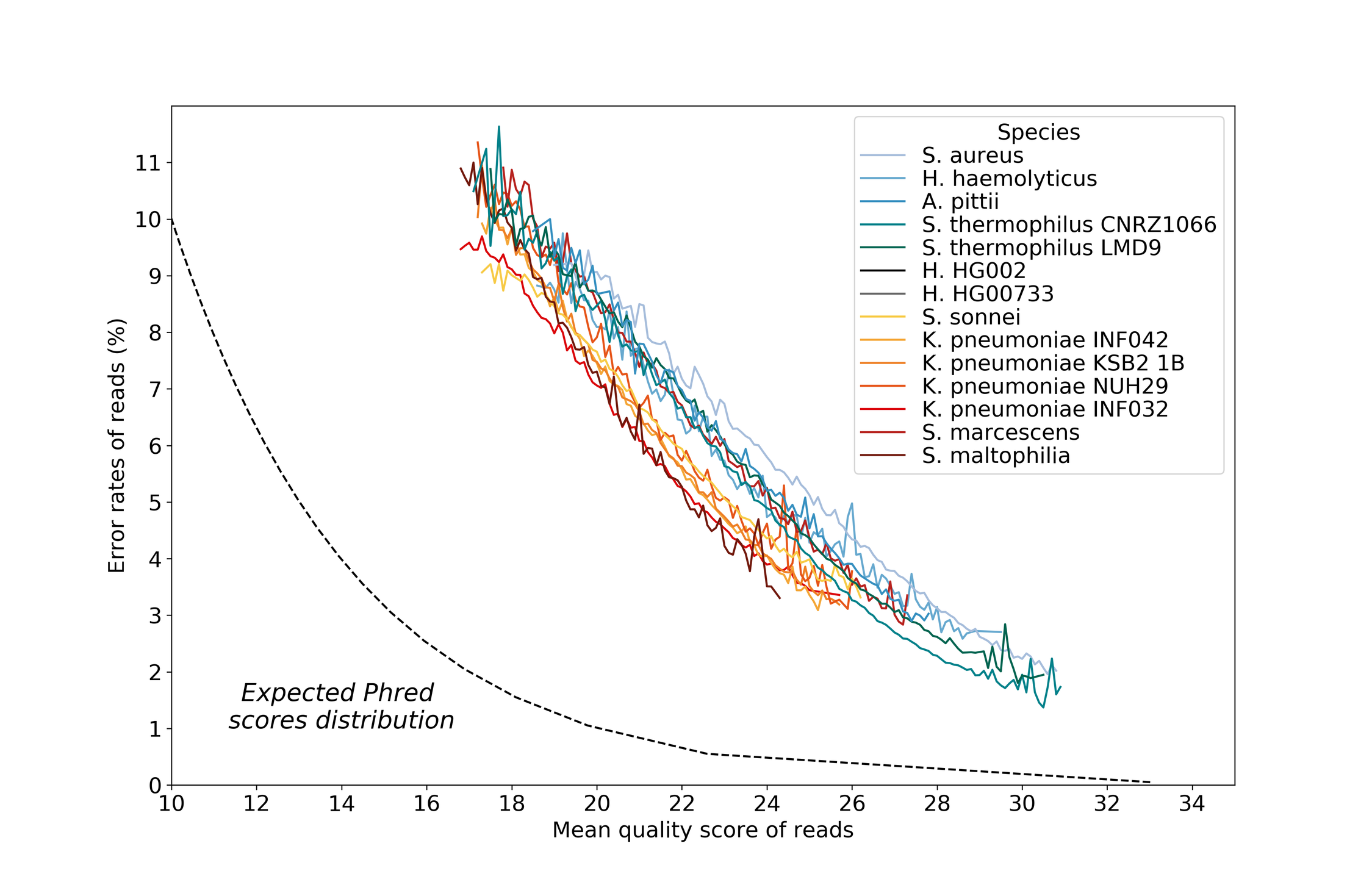

Supplement: S4 Fig — Quality scores are rounded to the first decimal value. The dotted black line represents the expected Phred score relationship between quality score and error rate, other lines represent results obtained for our studied species. Results were computed all bacterial aligned reads, and on 100,000 aligned reads for each human dataset. Only values supported for at last n reads are shown (n = 10 for bacterial data, n = 10, 000 for human data). (TIF) [file pone.0257521.s004.tif]

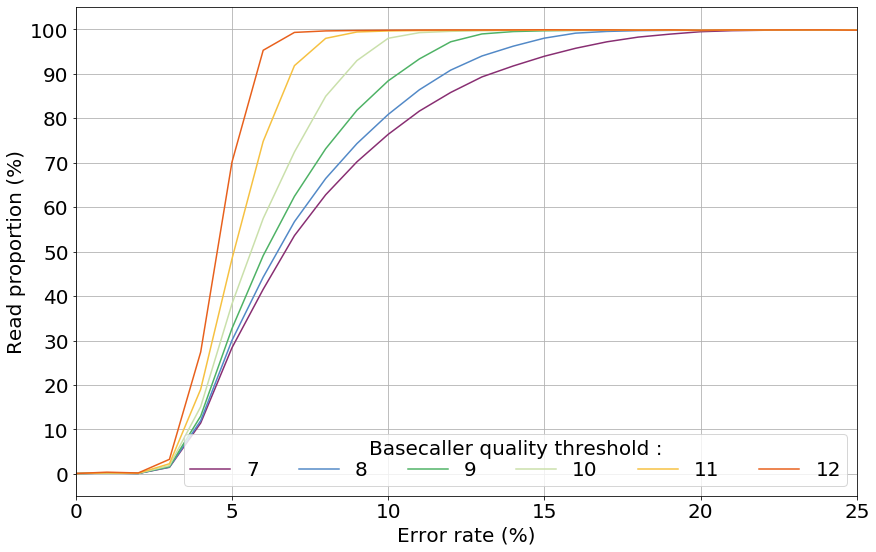

Supplement: S5 Fig — Results are computed on bacterial datasets. (TIF) [file pone.0257521.s005.tif]

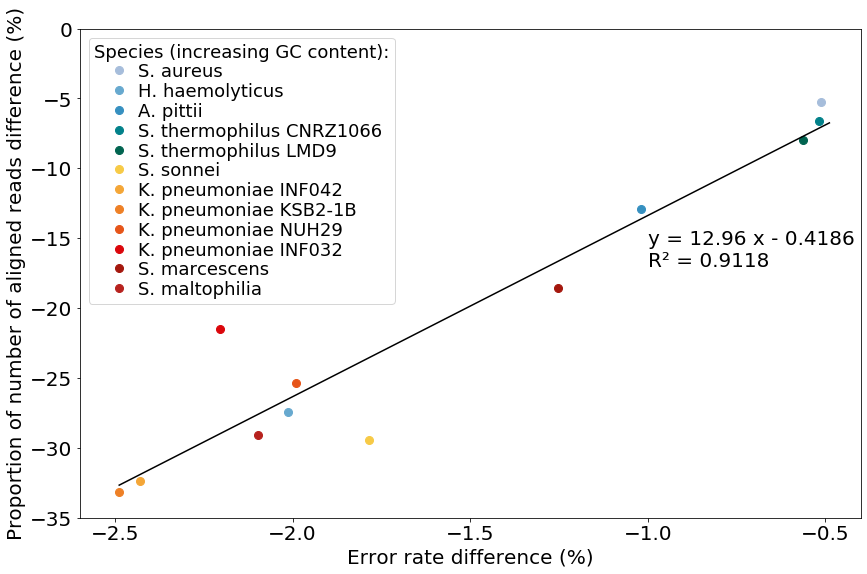

Supplement: S6 Fig — Read number loss is computed as the difference in number of reads between thresholds 7 and 10, divided by the number of reads for threshold 7. Error rate loss is computed as a simple difference in error rates between the two thresholds. Results are for bacterial datasets (colored dots). The black solid line shows linear regression. (TIF) [file pone.0257521.s006.tif]

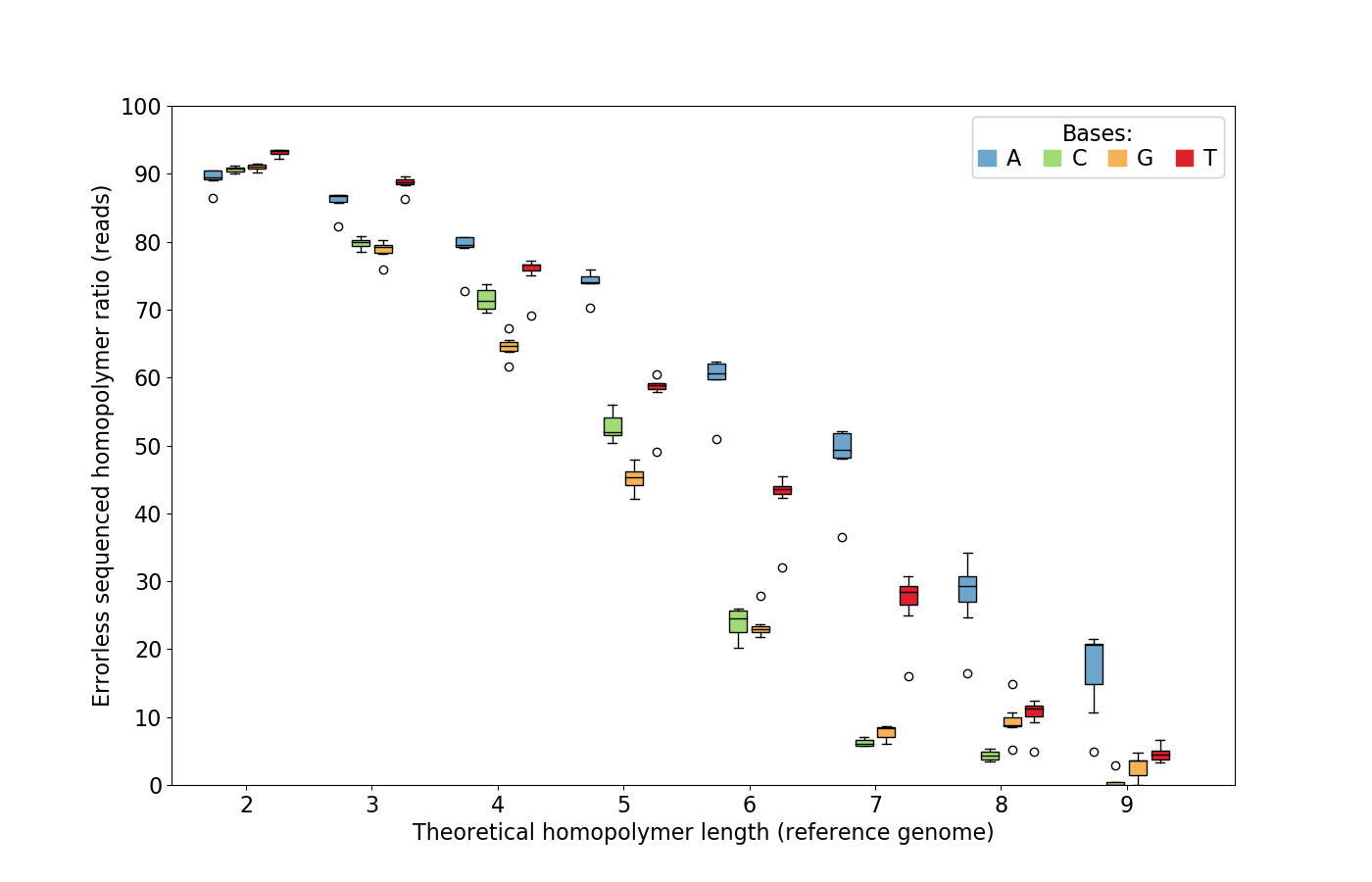

Supplement: S7 Fig — The base of the homopolymer does not strongly influence its sequencing accuracy, for length 2. However, for higher length, A- and T- based homopolymers are better sequenced than C- and G- ones. This trend is similar for low-GC bacteria and for human datasets. (TIF) [file pone.0257521.s007.tif]

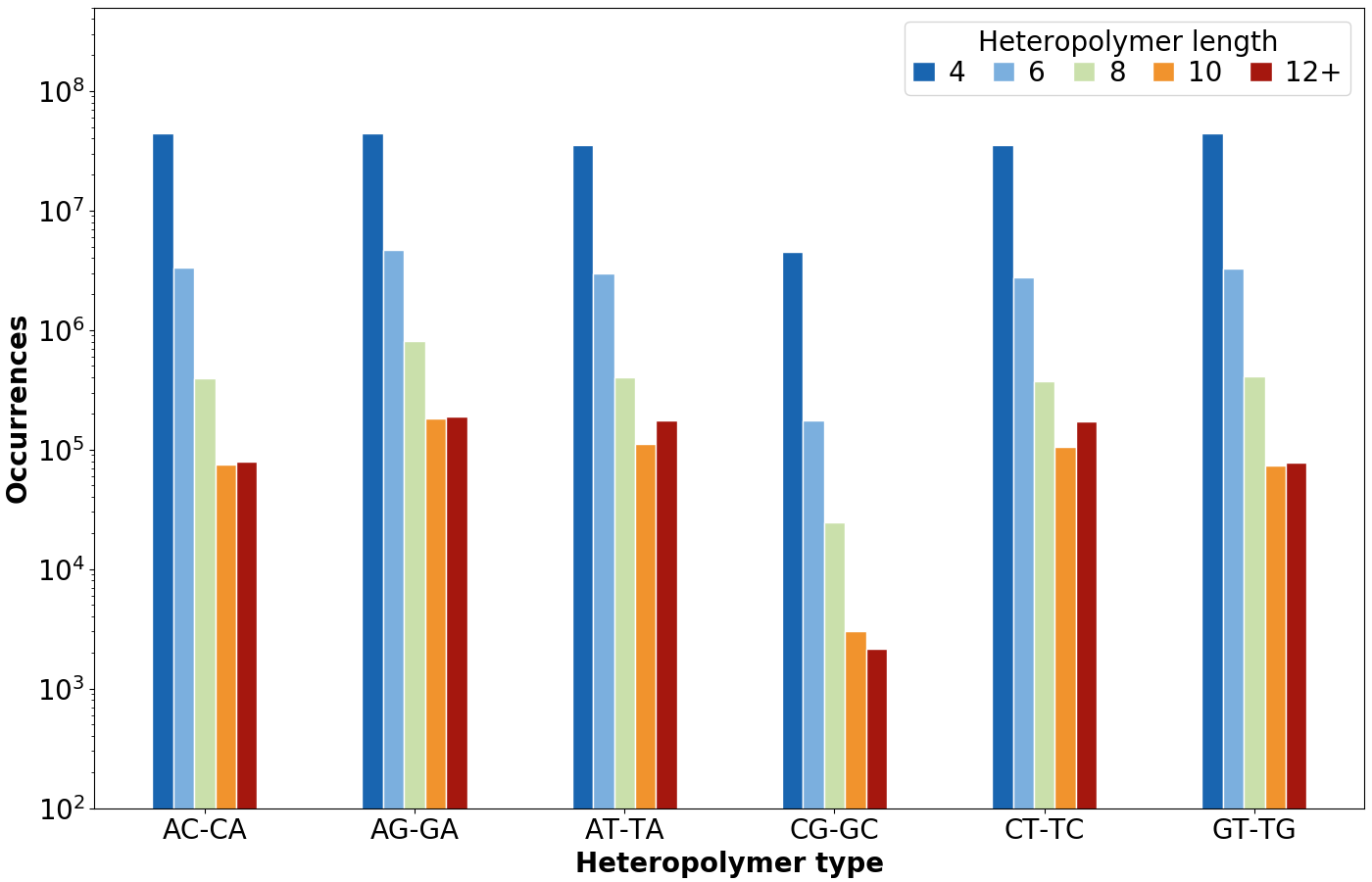

Supplement: S8 Fig — Symmetric dinucleotides (e.g. AC and CA) have been pooled. The scale is semi-logarithmic. (TIF) [file pone.0257521.s008.tif]

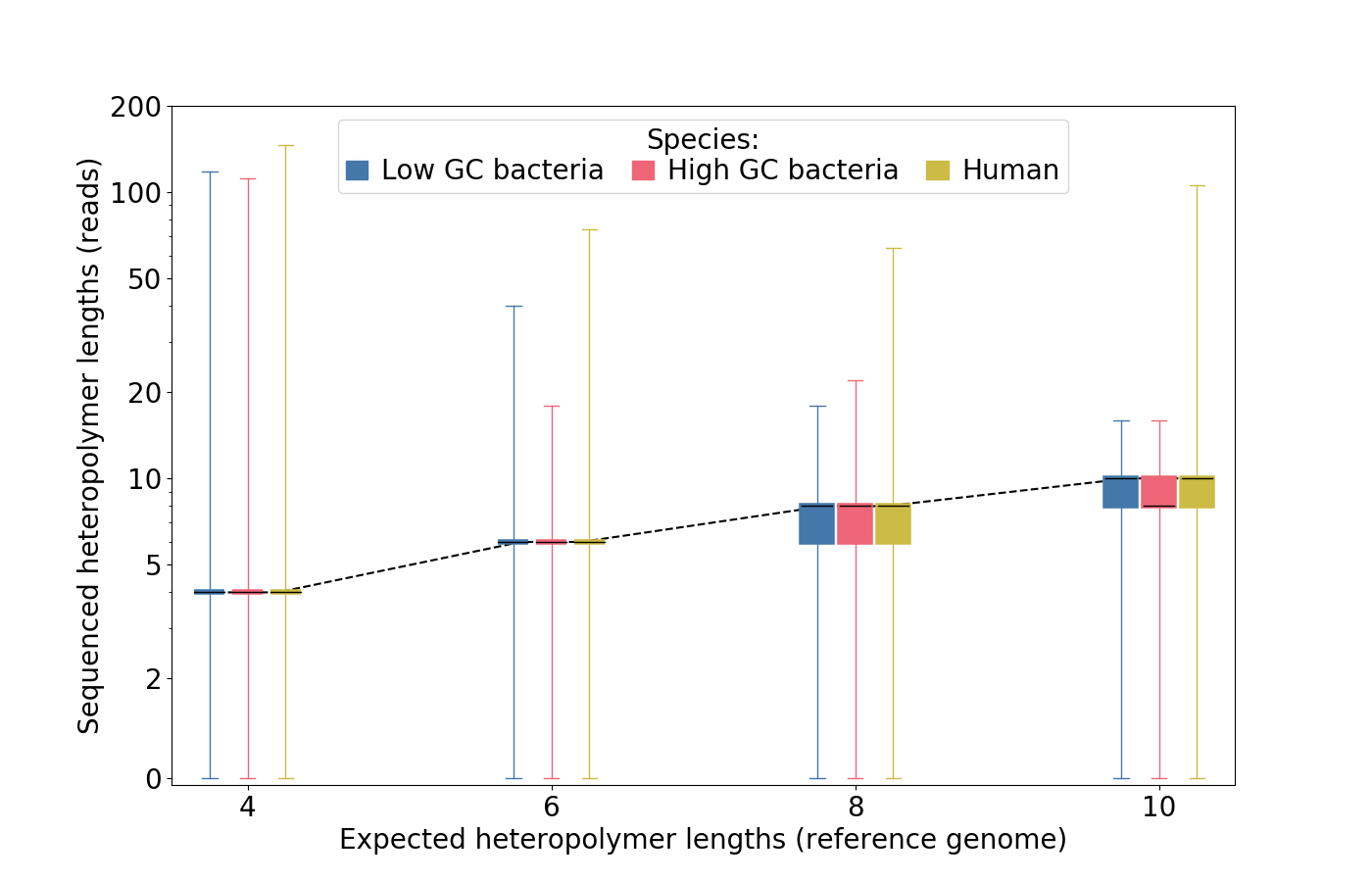

Supplement: S9 Fig — Species grouped in 3 categories: low, high GC content, and human. Scale is semi-logarithmic. Dotted line represents expected length. (TIF) [file pone.0257521.s009.tif]

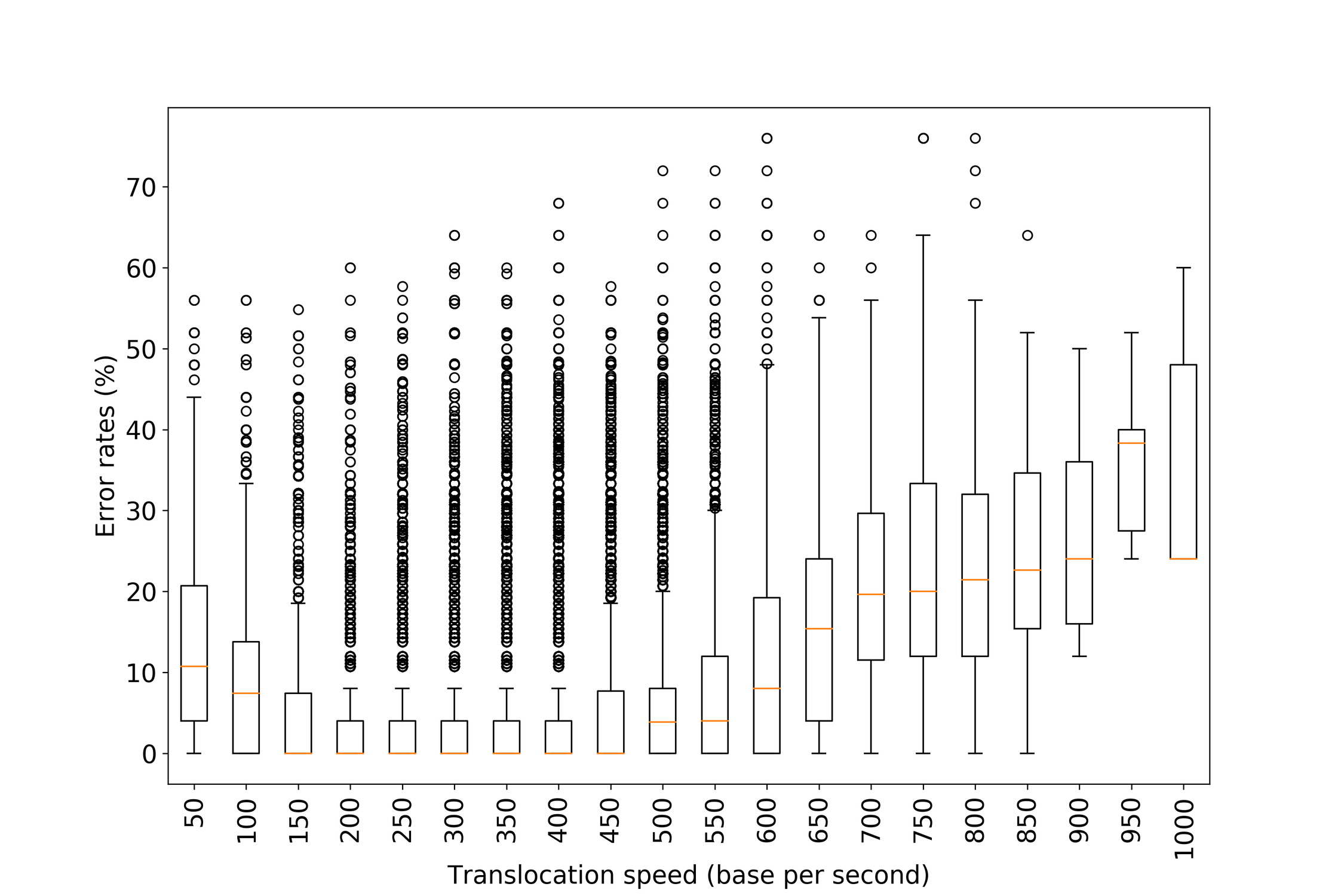

Supplement: S10 Fig — Computed on sliding windows of length 25 bases. (TIF) [file pone.0257521.s010.tif]

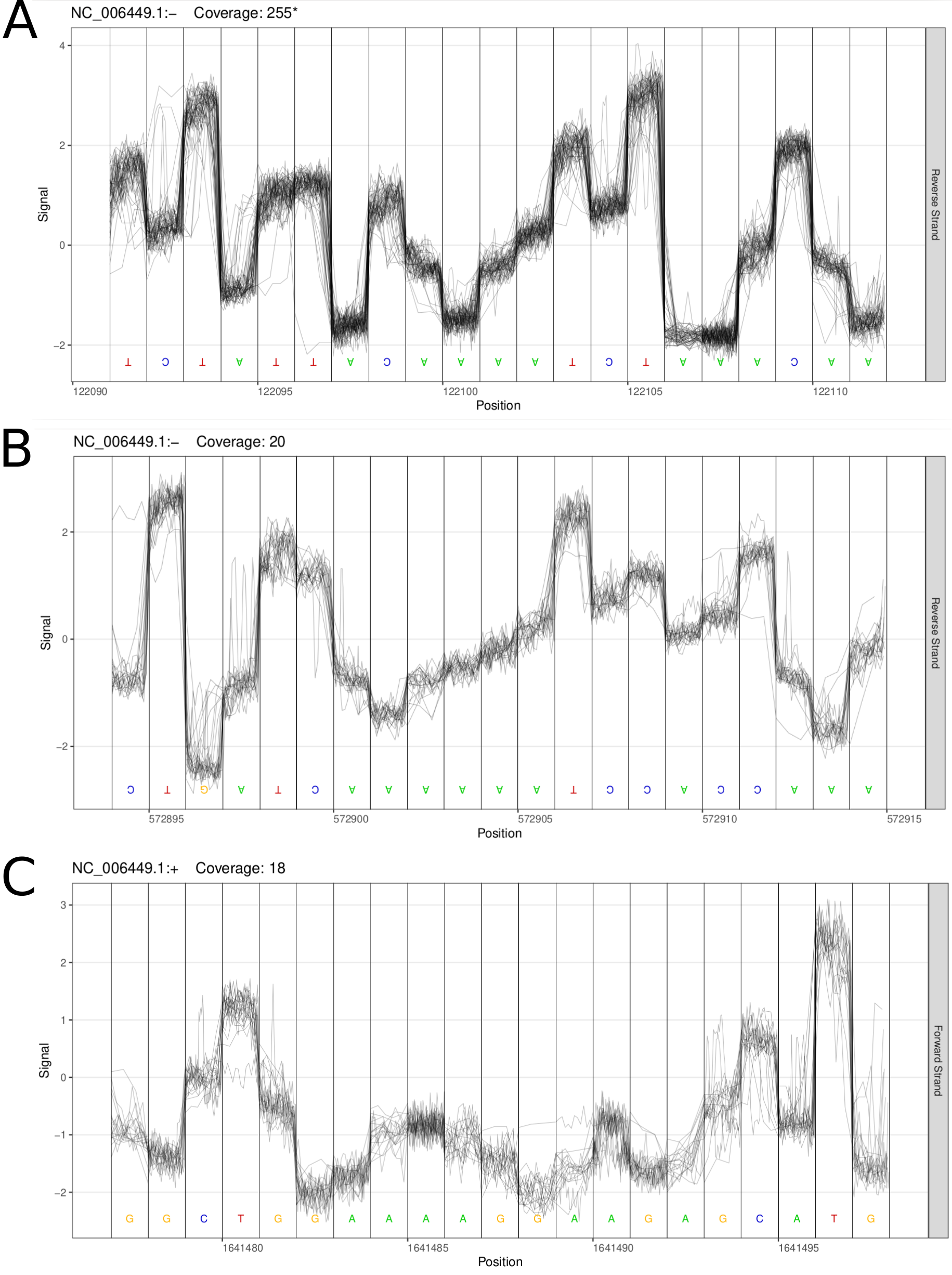

Supplement: S11 Fig — A- Tombo visualisation of 4-bases A homopolymer (centered). The homopolymer is rather short, and the associated signal is quite well segmented, which enables to easily delineate each base of the homopolymer. B- Tombo visualisation of 6-bases A homopolymer (centered). The homopolymer is longer than in A-, and the associated signal is harder to segment, which complicates the separation between each base. C- Tombo visualisation of 4-bases A homopolymer. The homopolymer is surrounded by G bases, for which the signal value is closer than for A- where the homopolymer was surrounded by T bases. This imply less variation in signal values, thus resulting in a more blurred signal. (TIF) [file pone.0257521.s011.tif]
