# Supplementary material for: Sequencing DNA with nanopores: Troubles and biases
Source: PLoS One. 2021 Oct 1;16(10):e0257521. doi: 10.1371/journal.pone.0257521 (PMC8486125; doi:10.1371/journal.pone.0257521)
Supplement: Attachment — Submitted filename: Rebuttal_letter.pdf [file pone.0257521.s013.pdf]

Dear editor and reviewer,

Thank you for your comments and critical assessment of our work. Here below we address the points raised by the reviewer. We have updated the manuscript accordingly and provied a version with the corrections highlited in addition to the revised version. For some points, new figures were computed, and have been added to the manuscript as supplementary data.

Sincerely,
Clara Delahaye
On behalf of all authors

1 Major issues **2**
1.1 Soft clipped bases 2
1.2 Possible local effects 2
1.3 Inverted repeats 3
1.4 Fast5 analysis 3

2 Minor issues **4**
2.1 Text corrections 4
2.2 Alignment of reads on reverse strand 5
2.3 CG heteropolymers - CpG 5
2.4 Wrong paragraph location 6
2.5 Figure 7 - Homopolymer abundance 6
2.6 Figure 9 - Homopolymer sequenced length, error in y-label 6

3 Other minor modifications **6**
3.1 Supplementary figure S1 6
3.2 Data availability - accession to our sequencing data 6

1 Major issues

1.1 Soft clipped bases

> **"Soft clipped" (Pg 4, lines 134, 135)**
> Here the authors state that reads that are over 50% soft-clipped are removed. However, if I understand the protocol correctly, reads have already been filtered if their Nanopore QC score is less than 10 (line 124). This suggests that there are reads with 50% soft-clipping that have QC scores 10 and up. This begs several questions that are central to the authors stated intent of understanding ONT error rates. Namely: How many reads were removed? If the read itself has QC >= 10, do the soft clipped bases also have high QC? If the soft clipped bases are the same between reads? (*ie.* assuming that at least two reads overlap - do the soft clipped bases align to each other (suggesting a true variation from the reference genome) or do they disagree (suggesting the sequencer is failing to accurately sequence anything, *ie* "noise")? If the soft-clipped reads are noise it would be very important to confirm that they have low QC - especially given the repeated failure of ONT to explain what Nanopore QC actually means.

Actually, reads with 50% of their bases soft-clipped, and having QC >= 10, are quite rare. For each bacterial dataset, we found mostly less than ten occurrences of such highly soft clipped reads. It represents less than 0.01% of reads (except for *S. marcescens* dataset for which it represented 5% ; around 0.5% for human datasets).
These soft clipped regions did not showed significant decrease of quality, being mainly around 20. It is not very clear for us why these huge read parts are not aligned while the whole read is tagged as aligned by the aligner, and the quality is not that bad.
For most of the datasets, there was not enough reads with more than 50% of their length soft clipped to be able to find overlaps. Nevertheless, for *S. marcescens*, we found that despite most of the soft clipped regions wont overlap, there are some occurrences of overlapping soft clipped regions. This suggests that soft clipped regions might indeed represent a true variation from the reference genome.
Concerning soft clips in general, as described in Supplementary figure S1, their quality shows a clear drop. While a certain part of the soft clipped bases can be attributed to adapters, the aligner clip additional bases, which means that the bases right next to adapters cannot be aligned with sufficient confidence.

We completed accordingly the subsection "Mapping reads to both strand of the reference" in Materials and Methods.

1.2 Possible local effects

> **"Possible local effects" (Pg 7, lines 233 - 253, Figure 1)**
> The authors seek to determine if there are local effects that can generate changes in error frequency. To accomplish this they scan the linearized bacterial genomes and plot the results and come to the conclusion that there may be no such effecst. However, there are several issues with this interpretation. First, the bacterial genomes are set to align at the beginning of the FASTA file - but that is an arbitrary position for circular bacterial genomes. While bacterial genomes do have a reference point that could be used for alignment (ie. replication origin (oriC)) that is not used here and the results are impossible to compare across species as a result. It would be much better to center the genomes at the oriC and then compare. Second, actual local effects may also be specific to a genome as not all the species have the same gene content and synteny. There are notable peaks amongst the noise -"with some possible local peaks" (line 246) - but to speak to the question of possible local effects these peaks would need to be interrogated (do they coincide with the oriC, rRNA, low-complexity regions?) and then compared across species.

Re-align bacterial genomes according to oriC

We used Ori-Finder (DOI: `https://doi.org/10.1186/1471-2105-9-79`)to locate replication origin, from reference genomes of our bacterial datasets. Overall, the oriC were very close to one end of the genomic sequence. We updated figure Fig1 accordingly. The figure remains very close to the previous version, with a stable error level.

Content of local peaks
Note first that the error variation is not high (standard deviation around 0.1%). Even for peaks, the difference hardly exceeds 1%. Nevertheless, we have studied the sequences for peaks above 3 standard deviation around the median. These sequences present no particular pattern, neither from the point of view of complexity, GC content, or type of annotation (coding mostly for proteins).

We corrected the figure Fig1, and completed the associated paragraph in corresponding subsection in Results.

1.3 Inverted repeats

> **"Inverted repeats" (Pg. 15, lines 551-555)**
> If I understand the work being referenced, these were very long inverted repeats produced from a CNV mutation. The absence of detection from these genomes is not indicative of a corrected basecalling method as these CNVs may not be present within these genomes. Unless the authors observed inverted repeat CNVs in these bacteria using the latest version of the basecaller, and observe CNV sequencing failures using a previous version of the basecaller it seems unwarranted to state that a solution has been produced.

Indeed, we had performed our study on small inverted repeats, and not on very long ones as presented in the cited work. We were not able to retrieve such long repeats (more than several kilo-bases) in our studied genomes, thus we cannot state on a potential improve of the basecaller for this point.

We preferred to remove the associated paragraph in our manuscript.

1.4 Fast5 analysis

> **"Fast5 analysis" (Pg. 16, lines 582, 583)**
> The authors state that because few Fast5 files are publicly available they are focusing on the post base-calling FASTQ files. While I am sympathetic to the lack of Fast5 file support in the public repositories - it appears that the Fast5 files are available for the data sets the authors have chosen to analyze. As several researchers have already shown (and as stated by the authors) analysis of the Fast5 files allows for the identification of changes in translocation rate. Since this would generate deletion errors (which are the primary contributor to error rates) it seems appropriate to investigate these in this case. Furthermore, an analysis of the Fast5 files would enable the authors to identify correlations in changes in error rate with the sequencing process itself.

We completely agree with the interest of out additional study on the raw electrical signal from fast5 files. We have worked on this point and improved our manuscript
In order to complete our error profile study, we conducted some additional analysis on the raw electrical signal from fast5 files. We completed the paper to include the following points (we added a subsection in Materials and Methods, completed the first paragraph in Results, as well as the one concerning Homopolymers, and added a subsection in Supplementary data with several figures).

To study the electrical signal from fast5 files, we used the tool Tombo from ONT (`https://github.com/nanoporetech/tombo`), more precisely its "resquiggle" algorithms. It needs the fast5 files containing the base-called reads (from fastq files) and a reference genome. In a nutshell, Tombo segments the raw signal and "map" it to the reference genome.
Thus we get, for each reference base, an estimation of the signal, *i.e.* the number and the value (normalized by Tombo) of all sampled electrical current. Knowing the rate at which the electrical is sampled, 4.000 samples per

second according to ONT, we computed the translocation rate, *i.e.* the speed at which bases go through the pore during sequencing. Given a stretch of N bases sequenced, a number of electrical signal E sampled at rate R, the translocation speed of bases S can be computed as: $S = \frac{N}{E} \times R$. For current chemistry, bases are supposed to translocate at an approximately constant rate of 450 bases per second.

Analysis of homopolymeric patterns

As homopolymeric regions are one of the most error prone sequences with nanopore sequencing, we started our investigation with these patterns.

First, our results show that the electrical signal is highly impacted by the surrounding bases, as stated by Nanopore. In the context of homopolymers, this results into (1) an usually well segmented signal, both in terms of duration and value, for homopolymers of length $\leq$ 5 bases long, thus being mostly well sequenced, unlike (2) longer homopolymers for which the current is far less influenced by surrounding bases, which results in a harder to segment signal, making it harder to correctly assess the homopolymer length.

Moreover, it seems that the level of the electrical current is a quite good indicator of the sequenced base. It appears that signal values can be roughly ordered along the sequence in the following way: $signal(T) \geq signal(C) \geq signal(A) \geq signal(G)$. Clearly, it will be harder to distinguish for instance a transition A-G than a transition A-T. In practice, this also also impacts the way homopolymers are sequenced: an homopolymer of A, surrounded by T bases, will be better sequenced than an homopolymer of A surrounded by G bases, as the difference in signal level is more pronounced in the first case.

Translocation speed for deletion errors

Deletion errors is the other main source of errors in Nanopore sequencing, we studied the translocation speed associated to these errors. We computed the translocation speed corresponding to deleted bases in reads, as well as the ones corresponding to surrounding bases (10 bases before and after), for deletions of at least 5 bases. We found that these errors were associated to a high increase of translocation speed: $\geq$ 1,200 bases per second while surrounding bases display a translocation speed around 460 to 520 bases per second.

Correlation between translocation speed and error rate

Finally, we gathered error rates associated to different translocation speeds. While the current expected speed (around 450 bases per second) is associated to low error rates (median below 5%), a higher translocation speed lead to a dramatic increase of error rate (above 20% for speeds over 650 bases per second). As mentioned above, it is to be linked with deletion errors. More surprisingly, a slow base translocation is also associated to higher error rate (5 to 10% for speeds below 150 bases per second), this time being mainly associated to insertion errors.

2 Minor issues

2.1 Text corrections

> Abstract - "Oxford Nanopore Technologies" should be used once before being abbreviated to "ONT".
> Line 5 - Replace "proposes" with "that uses"
> Line 160 - Replace "couple" with "pair"
> Line 258 - Replace "in average" with "on average"
> Line 278 - Replace "errors" with "error"

These have been corrected in the revised manuscript.

2.2 Alignment of reads on reverse strand

> **Alignment of reads on reverse strand**
> Line 140 - Very interesting insight. Did this change the observed error rates?
> Line 290-292 - Unclear if the observations did change when you used reverse sequence or they did not change. (See comment for Line 140, above).

(We completed the corresponding sections in "Materials and Methods" and "Results" with the following:)
The observed error rate was not noticeably different when taking into account strand detection.
Nevertheless, while the global error rate is hardly changed, the information contained in the alignment is totally different. Consider a short example, with a read $R = GCCAAGACCT$ aligned on the corresponding part of the genome $G = ACGTCATTGC$:

```
G        ACGTCATTGC            rev(G)  GCAATGACGT
         | |||  |  ||                  ||  |  |||  |
rev(R)   AGGTCTTGGC            R       GCCAAGACCT
```

The example on the left shows what most alignment tools currently do: the read is reverse-complemented and aligned on the forward strand of the reference genome. On the right, the read is unchanged and the genome is reverse complemented. Here for simplicity we suppose the aligner performs an exact alignment and does not use heuristics.
In both cases, the error rates are the same (*i.e.* 30%). However, the errors themselves are different: in the left case errors are C/G, A/T and T/G, while in the right case errors are A/C, T/A and G/C (X/Y denotes a substitution of a base X in the genome by a base Y in a read). Thus, the impact of the chosen alignment method for error profiling is crucial.

In practice, when we did not apply the specific handling of reverse-mapping reads (as describe above), our findings were very close to those of the other researchers (*i.e.* transitions were three times more frequent than transversions). Whereas when applying this handling, our observations were impacted: this time, only A to G substitutions were impacted and three times more frequent.

2.3 CG heteropolymers - CpG

> Line 494 - "CG heteropolymers for which the error rate is much higher." This is a very interesting result and yet it seems to be unaddressed. Is the higher error rate of CG in human data indicative of the CpG rates?

In order to determine whether the high error error rate of CG in human data were related to the CpG island, we specifically computed the error rates on CpG islands for human datasets. To do so, we based on an UCSC's database of known CpG islands in human genome (`http://hgdownload.cse.ucsc.edu/goldenpath/hg38/database/cpgIslandExt.txt.gz`). We computed the error rate of regions of reads that aligned on these regions.
We found that the error rate was in fact really close to overall error rate, *i.e.* around 6 to 8%, and not as high as what has been observed in CG heteropolymers in human genome. Thus, it seems that the higher error rate concerning CG heteropolymers could not be related to CpG regions.
We added a short paragraph describing this result in the corresponding Result section.

2.4 Wrong paragraph location

> Line 551 - Not clear why this paragraph is under the 'Perfect Kmer section', potentially a formatting issue?

Yes, it was a mistake. This paragraph has been moved to the next section 'Sequence-specific errors'.

2.5 Figure 7 - Homopolymer abundance

> **Figure 7 -** From this figure it is difficult to identify if the homopolymers are causing the observed abundances in sequencing errors or if it is the frequency of these homopolymers in the genome.
> By which I mean, are A/T homopolymers more error prone or just more common? Does normalizing by frequency in the genome (or frequency of reads, as in Figure 8) remove the apparent difference between A/T and C/G?

The Figure 7 actually represents the abundance of the homopolymers (according their length and base) in the reference genome, and is not about sequencing errors related to homopolymers. The supplementary figure S7 (similar to Figure 8) gives insights about which homopolymer categories are more error prone than others.
Exploiting both figures, we can see that A/T homopolymers are globally more common (Figure 7 ; except for 2-bases long homopolymers in bacterial data), and that A/T homopolymers are indeed better sequenced than C/G homopolymers, independently of their frequency (Supplementary figure S7 ; again except for 2-bases long homopolymers).

2.6 Figure 9 - Homopolymer sequenced length, error in y-label

> **Figure 9 -** The plot is difficult to understand, is the Y-axis incorrectly labelled?

Yes you are right, the Y-axis was indeed incorrectly labelled with the same label as Figure 8. We modified the figure with the correct Y-axis label is *"Sequenced homopolymer lengths (reads)"*.
The plot represent the difference between sequenced (in reads) *versus* expected (in reference genome) length of homopolymers.

3 Other minor modifications

3.1 Supplementary figure S1

The bottom part of this figure was erroneous: we forgot to apply the -33 to quality scores for these values, thus the y-axis was wrongly labeled. This figure is now corrected.

3.2 Data availability - accession to our sequencing data

The link provided would not work anymore ; we provide a new one that will be long-term available.
